# The Curability of Pulmonary Phthisis

**Published:** 1885-08

**Authors:** 


### BOOK REVIEWS.

THE CURABILITY AND TREATMENT OF PULMONARY PHTHISIS *By* S. JACCOUD, *Paris.* *Translated by* MONTAGUE HALLOCK, M. D., *London.* *New York:* D. APPLETON & CO.; *Chicago:* JANSEN, MCCLURG & CO. *1885.*

This book is the substance of a course of lectures by Professor Jaccond, delivered during the winter of 1881 and 1882. In the preface the author states that he has been led to publish the work, both on account of the original character of certain pathological views which he holds, and also by the novelty of his conclusions and methods of treatment.

The first chapter is in part devoted to an attempt to prove that phthisis is curable—a position which will scarcely be questioned, I presume, by any one familiar with the American literature of the subject. He believes in the unity of tubercle but admits a duality of form, which, after all, consists only in quantity of tuberculous matter.

The conditions which influence the curability of the disease are considered in the second chapter. In studying these, he divides the cases into three (3) groups.

1. The hereditary.
2. The innate.
3. The acquired.

The first he thinks if less curable, is still amenable to treatment, but this treatment should be commenced in infancy and continued to adult life, if indeed adult life is reached. Children should not be allowed to nurse phthisical mothers, and should not be allowed to live in the family with phthisical patients.

The author firmly believes in the communicability of the disease, but as these lectures were delivered before the discovery of Koch, he is unable to form an opinion as to the agent of communication. Rightly, as all will agree, he attaches the greatest importance to good nutrition as a prophylactic measure.

By innate phthisis is meant that form of the disease "observed in descendants of those who, though not tubercular, are weakened by scrofula, cachectic diabetes (?), alcoholism, or simply by bad hygienic conditions." Precisely how phthisis exists in a latent form in these persons the author explains only by the general statement that "tuberculosis is the common result of all forms of constitutional deterioration, either in the family or in the individual."

The innate form, he considers more amenable to treatment than the hereditary.

The acquired phthisis he again divides into two groups:

1st. Those cases resulting simply from mal-nutrition.

2nd. Those consequent upon some constitutional defect that is secondary to some other disease.

Of all forms of phthisis, those depending upon simple mal-nutrition are the most hopeful under proper treatment.

We cannot follow the author through a somewhat lengthy discussion of the etiology and the conditions of curability, but the reader who has patience to read this chapter will find many suggestions of interest.

The lectures upon climate are especially interesting—reference being made to the principal stations in Europe and Northern Africa, while no mention, or only a brief reference, is found to localities in America.

The style of the work is diffusive and in some parts obscure or contradictory. The translator assumes on the part of the reader a pitiable ignorance of scientific terms, thinking it necessary to explain the meaning of such expressions as "*histogenetic*," "*hydrotherapeutics*," "*epigenesis*," "*heteromophism*," etc., etc. He certainly desires to be understood.

